# Web-based ecological evidence entry form enables consistent, accessible extraction and visualization for synthesis applications

**DOI:** 10.1111/csp2.13278

**Published:** 2025-01-23

**Authors:** Caroline E. Ridley, Casey Hansen, Peter Byrley, Tara Greaver, S. Douglas Kaylor, R. Byron Rice, Kate A. Schofield, Andrew Shapiro

**Affiliations:** 1 U.S. Environmental Protection Agency, Office of Research and Development, Center for Public Health and Environmental Assessment, Research Triangle Park, North Carolina, USA; 2 Oak Ridge Institute for Science and Education Fellow at U.S. Environmental Protection Agency, Office of Research and Development, Center for Public Health and Environmental Assessment, Research Triangle Park, North Carolina, USA; 3 U.S. Environmental Protection Agency, Office of Research and Development, Center for Public Health and Environmental Assessment, Washington, District of Columbia, USA

**Keywords:** assessment, conservation, controlled vocabulary, data visualization, ecology, environmental management, evidence, synthesis, systematic review, web-based content management

## Abstract

Applying scientific evidence to conservation, environmental management, and policy-making improves outcomes. When synthesizing existing evidence, substantial resources are required to access and read scientific publications and extract and analyze decision-relevant information. To improve this process, we developed a free, publicly available, web-based evidence entry form tailored to extract information about cause-effect relationships from ecological publications. The form enables storage, retrieval, reuse, and visualization of qualitative and quantitative ecological and environmental evidence extracted from publications. Evidence can be analyzed for a wide range of synthesis purposes (e.g., causal assessments, hypothesis testing) and approaches (e.g., rapid reviews, meta-analyses). The database schema underlying the form logically relates information about (a) a publication, (b) its experimental design(s), and (c) reported cause-effect relationships. An ontology of controlled terminology enables consistent extraction and characterization of causes and effects across users, facilitating evidence reuse. Future capabilities include customization of terminology and incorporation of study quality information.

## 1 | INTRODUCTION

Scientific evidence is essential for effective conservation, environmental management, and policy-making, yet many challenges exist to generate, gather, evaluate, synthesize, communicate, and apply evidence (Cooke et al., 2023; Crequit et al., 2020; Fisher et al., 2020; Lubchenco et al., 2015; Sutherland et al., 2004; Walsh et al., 2019). For those who rely on existing evidence to inform environmental decision-making, after published scientific articles and reports are acquired (a challenge in itself), additional and substantial resources may be needed to read, extract, and analyze the evidence contained in those publications. There are many dedicated efforts to enhance the value and efficiency of the process of bringing existing evidence to management and policy (Crequit et al., 2020; Fisher et al., 2020; Haddaway et al., 2017; Matthews, 2021; Nakagawa et al., 2020).

At the same time, a related movement for transparency, reproducibility, and FAIR principles (Findability, Accessibility, Interoperability, and Reusability) in science is going strong (Cannon et al., 2022; Nakagawa et al., 2020; Wilkinson et al., 2016). Conservation funding institutions often have requirements for transparency to ensure that grants produce reliable results in a cost-effective manner. Similarly, as scientists within the US federal government, we are keenly aware of expectations and requirements to develop systems and workflows that are transparent and that serve the public.

Within the US EPA’s Office of Research and Development (ORD) and Center for Public Health and Environmental Assessment (CPHEA), we routinely produce evidence syntheses that support many kinds of environmental decision-making. These include syntheses that inform national-scale decision-making (e.g., Integrated Science Assessments [ISAs] that form the scientific foundation for the US National Ambient Air Quality Standards), state-level decision-making (e.g., syntheses and reviews supporting US state-led Water Quality Standards), and other local and community decision-making to protect and restore human health and the environment. We also develop tools and approaches for evidence synthesis that support our work, as well as more widely benefit others undertaking evidence syntheses (e.g., rapid evidence assessments, systematic reviews, meta-analyses).

One of these tools is the Health Assessment Work-space Collaborative (HAWC). HAWC is a web-based content management system that supports systematic reviews and various types of analyses of scientific literature (Shapiro et al., 2018). HAWC is an open-source software application built using a Python web framework called Django (Django, 2023). Currently, HAWC is used by scientists at institutions including the US EPA, the National Institutes of Health, and the World Health Organization to aid in human health and environmental assessments of pollutants. It has not been used for aiding ecological assessments, because a suitable way to capture aspects of ecological studies that are essential, the experimental setting (e.g., field, greenhouse), habitat, climate, level of biological organization (e.g., from sub-organismal to ecosystems), particular statistical measurements, was not available until now.

We are expanding the utility of HAWC by developing a new form within it to facilitate the extraction of information about cause-effect relationships from ecological and environmental science publications. As with previously existing forms within HAWC that were designed separately and specifically for the extraction of bioassay, epidemiology, and in vitro studies, our goal with this new form is to enable the extraction, storage, retrieval, reuse, and visualization of qualitative and quantitative evidence from publications. Evidence can be exported and analyzed for a range of synthesis applications, including causal assessments and hypothesis testing. In this article, we describe the form development process, along with its features, capabilities, and limitations. We end by discussing potential future features and applications, especially related to conservation science.

## 2 | METHODS

### 2.1 | Development of the form

We started by cataloguing examples of evidence extraction forms outside of HAWC that were known to the authors from the fields of ecology and environmental science. None of these existing forms have the wide availability and application that was our goal for the HAWC form, but it was useful to identify common organizational features and functionalities among them. We examined both web-based forms (e.g., the aquatic-focused EcoEvidEx https://ecoevidex.essolutions.com.au/) and spreadsheets that were developed for specific projects but existed only on desktops of individuals or teams. We also gathered examples from related disciplines. For example, the US EPA ECOTOX Knowledge-base (https://cfpub.epa.gov/ecotox/) is populated using an evidence extraction form for ecotoxicology studies, and HAWC includes an evidence extraction form for epidemiology studies.

We developed a new module within HAWC for data extraction and visualization of ecological and environmental data. Simple forms automatically generated by the HAWC application’s underlying Django software were initially used to evaluate the usability of the data schema defined in the new module. A 5-person user group tested this preliminary form by extracting evidence from five articles (Supporting Information S1); each article was extracted by two people. These articles consisted of observational and experimental studies, field and chamber studies, studies with qualitative and/or quantitative responses, and studies involving various media (e.g., land, water, and/or air). After these test extractions, the user group discussed possible revisions to the form to improve evidence extraction consistency and user experience. In addition, a new database schema was created to more closely match how data are logically related in these studies (Supporting Information S2).

Once the user group feedback had been aggregated, a software engineer developed the second version of the form with an updated database schema and a custom user interface (UI) integrated into the HAWC site. The same user group tested the form again by extracting two new articles each (Supporting Information S1), which resulted in revisions to improve user experience and to ensure that the form fields, field pick lists, field order, and help text supported some specific desired outputs. These outputs include evidence tables for ISAs and visualizations for the effects of in-stream nutrients on biological communities, which EPA ORD ecologists are interested in using HAWC to generate.

### 2.2 | Development of ontology and controlled terminology

We developed controlled terminology lists for fields of information in the evidence extraction form that were amendable (Supporting Information S3). Controlled terminology increases consistency and comparability of extracted information across study reviewers and reduces effort dedicated to data clean up, enabling faster results generation.

Most lists were straightforward to generate (e.g., list of US states, list of quantitative measures of association), however, the ontology and controlled terminology for “cause term” and “effect term” required additional effort. “Cause” in our form is also known as the independent variable, stressor, or treatment. “Effect” in our form is also known as the dependent variable, response, endpoint, or outcome. Our goal was a useful set of contemporary, scientific cause and effect terms organized in a logical way that we could apply to ongoing work at the US EPA to support decision-making. The list needed to capture the wide diversity of causes and effects (e.g., chemical, physical, and biological) that are a part of the US EPA’s mission to protect the environment and to include a way to extract information on a spectrum of specificity.

We began by considering existing ontologies and controlled terminology lists related to ecology and environmental sciences, as well as concepts and terms addressed in recent assessments published by US EPA ORD (Bennett et al., 2021; U.S, 2020). We drafted an initial set of nested terms, and then consulted with subject-matter experts to add to it and revise it. Throughout rounds of testing the form (see Section 2.1), we continued to add and revise terms. The final list, which is identical for the cause and effect fields of the form, consists of 1828 terms that may be further characterized in a free text comment field. We acknowledge that one correct ontology and list of terms does not exist, especially given evolution of language and different regional uses of scientific terminology. Potential exists for continued term additions (e.g., specific conservation interventions) and revisions via designated curators on a project-by-project basis.

## 3 | RESULTS AND DISCUSSION

**FIGURE 1:**
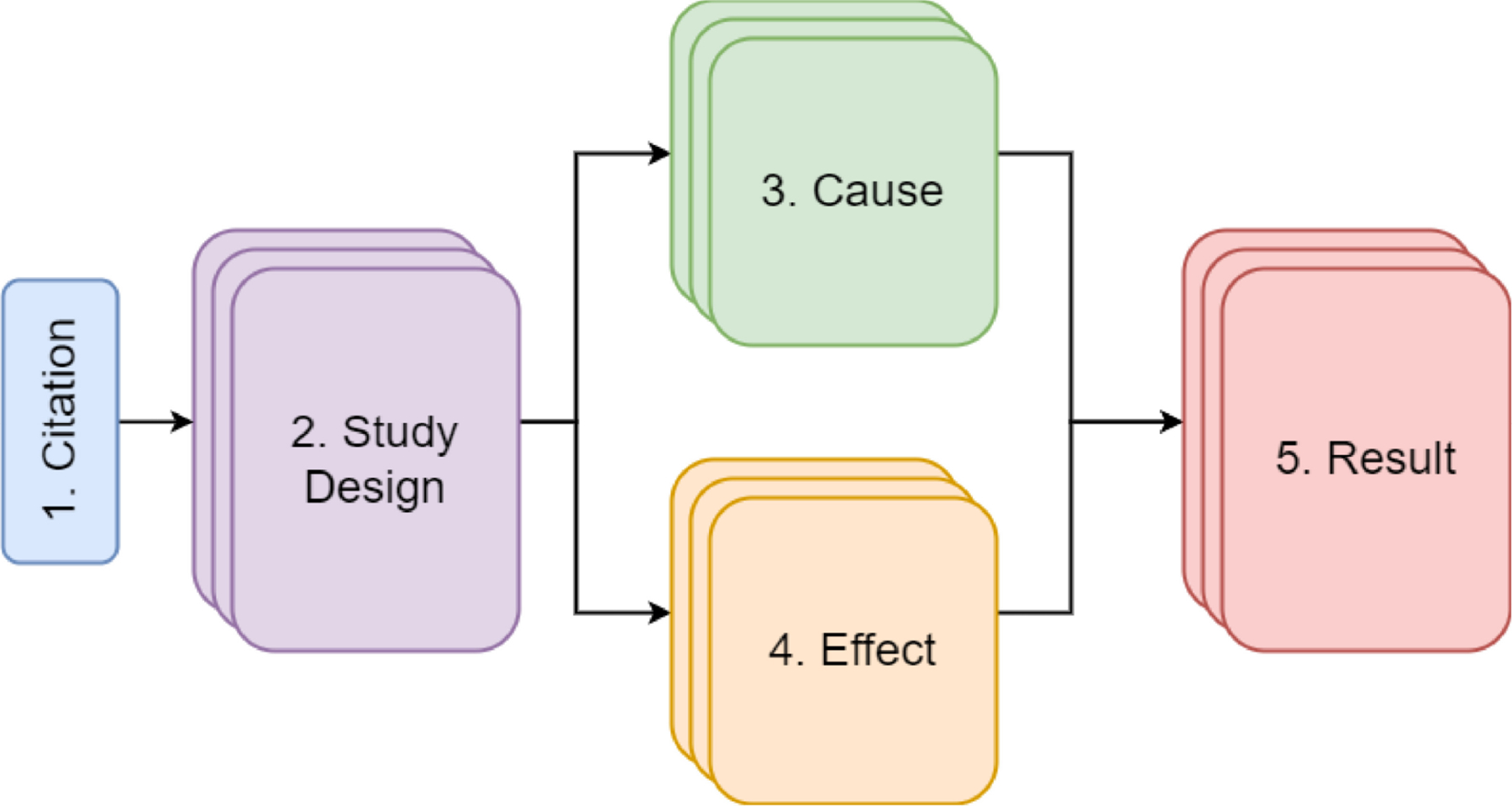
The HAWC ecological evidence entry form has five parts: Citation, Study Design, Causes, Effects, and Results. A Citation can have one or more Study Designs. A Study Design can be associated with one or more Causes and one or more Effects. A Result is made up of one Cause and one Effect that are selected by a user. Additional information about the relationship between a Cause and Effect completes the Result. A Study Design can have one or more Results. A case study assessment that has 14 citations can be found here: ORD Assessment Ecological Forms. To access the citations in this assessment online, click “Study list” on the left menu; each citation has a link that brings the user to additional bibliographic information, Study Designs, and evidence that has been extracted.

The form is organized into five parts that are connected by a database schema logically relating them (Figure 1; Supporting Information S2). Screen captures of the form can be found in Supporting Information S4. A spreadsheet of all fields in the form and their lists of controlled terminology can be found in Supporting Information S3. The five parts of the form are:

1. Citation—Bibliographic metadata about a publication is imported using a unique identifier from either PubMed (https://pubmed.ncbi.nlm.nih.gov/), the US EPA Health and Environmental Research Online (HERO) database (https://hero.epa.gov/), or manually entered by the user via uploading an RIS reference file.
2. Study Design—A user extracts information from the publication about the context and conditions under which a study or studies take place. One publication may be associated with one or multiple distinct study designs.
3. Cause—A user extracts qualitative and quantitative information about a cause variable in a study. Each distinct study design may be associated with one or multiple causes.
4. Effect—A user extracts qualitative and quantitative information about an effect variable in a study. Each distinct study design may be associated with one or multiple effects.
5. Result—A user associates a cause and an effect to create a result. Then, the user extracts additional qualitative and quantitative information about how a cause and effect are related to complete the result. A study design may have one or multiple results.

A case study of the current evidence extraction and visualization capabilities is demonstrated here: ORD Assessment Ecological Forms. This case study is a sample of studies that were included in a systematic review of the effects of nitrogen and phosphorus on chlorophyll *a* concentration in streams and rivers (Bennett et al., 2021).

**FIGURE 2:**
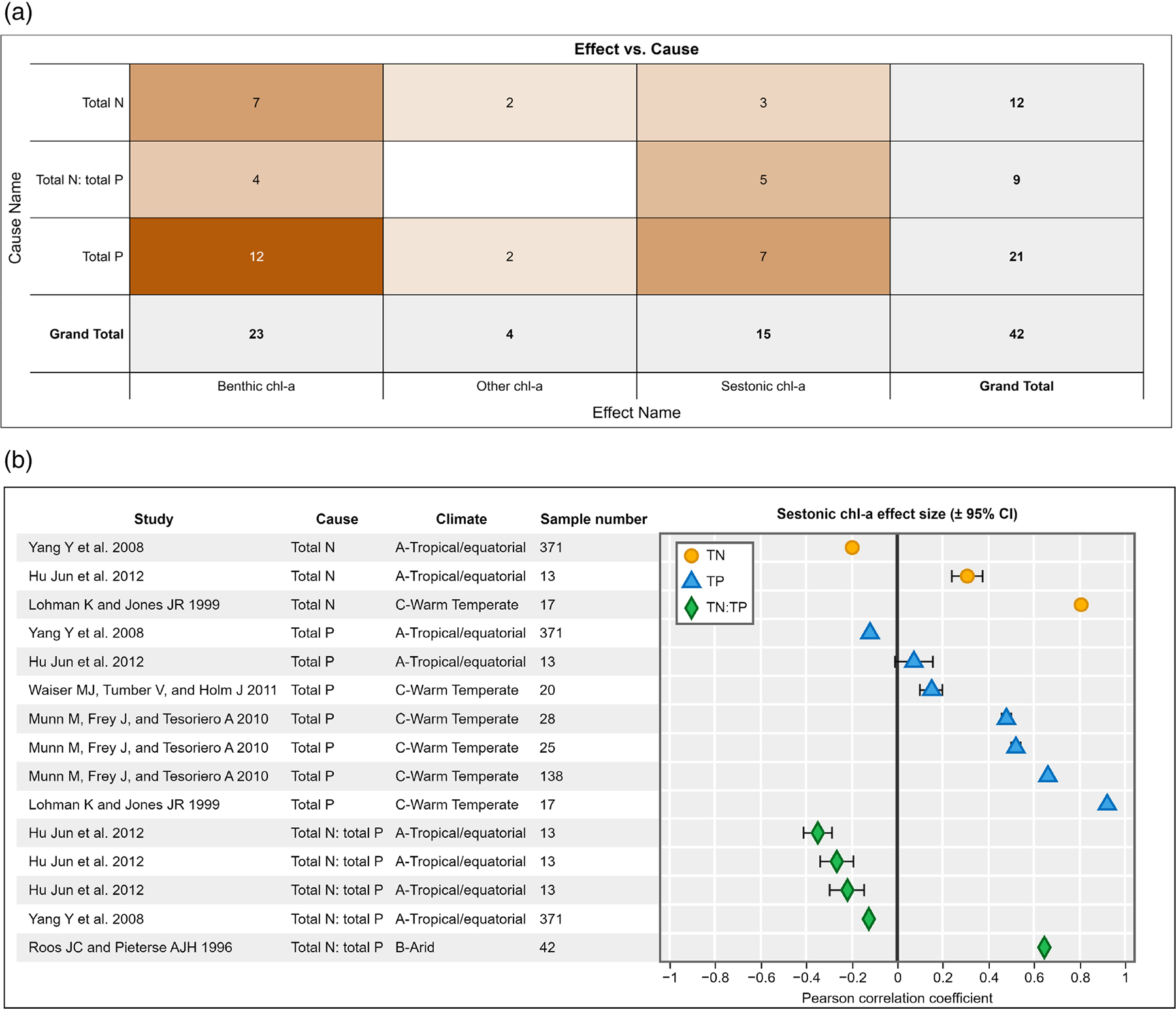
Examples of visualizations that can be produced from evidence entered into the HAWC ecological evidence form including (a) a standard heat map that counts the number of Results available for Cause-Effect combinations in an assessment. To access the dynamic version of the heat map online, visit ORD Assessment Ecological Forms and click “Endpoint list” on the left menu. The “Results” link in the table shows the standard heat maps with counts of Results as in Figure 2; the “Study summary” link in the table shows the standard heat maps with counts of studies. Custom forest plots can also be produced (b) which show quantitative effect sizes for a set of citations. Note that in this forest plot, several citations are associated with more than one Result. This happens because there is more than one Study Design for these citations. To access the custom forest plot online, visit ORD Assessment Ecological Forms and click “Visualizations” on the left menu and then click the “Forest plot” link in the table.

Several standard visualizations and an array of custom visualizations are associated with the HAWC form (Figure 2). Three standard heat maps visualize the abundance of evidence. The first standard heat map summarizes counts of Results that relate cause and effect terms; in the continuous color scale, darker shades indicate more abundant evidence (Figure 2a). The second and third standard heat maps summarize the counts of cause terms and effect terms, respectively, across all studies in an assessment (Supporting Information S5). All standard heat maps can be filtered by additional fields.

A user can also create custom heat maps and forest plots. Forest plots show point estimates and confidence intervals for a set of individual study results in the scale of a common effect measure (e.g., Pearson correlation coefficient). With HAWC’s visualization capabilities, a user can create, for example, a forest plot that shows the correlation coefficients for a set of studies in which different causes are shown with distinct colors and/or shapes (Figure 2b). Note that a summary effect size does not appear on forest plots generated by HAWC, because a meta-analysis model is required to generate one; data exported from HAWC could be used in separate analysis software to conduct meta-analysis and generate a summary effect size. All visualizations can be exported as an SVG, PNG, or JPEG file.

In practice, the form is used in the context of HAWC “assessments,” which are user-defined collections of studies with evidence relevant to answering a scientific question. Assessments can be initiated by anyone with a HAWC account and assessment roles are assigned by an assessment lead (who is generally the user initiating an assessment). Assessments can be categorized as private or public and this categorization can be changed throughout their life cycle. For example, assessments are often initiated as private and stay private through the development phase, then move to a public status once an assessment is completed and/or published. Once public, evidence can be retrieved by clicking on the corresponding link at https://hawc.epa.gov/assessment/public/. Evidence entered into the form is stored in the HAWC database.

A user may download evidence within a public assessment by first clicking on the “Download” button from the left-side navigation under a specific assessment. This takes the user to a new page where the desired evidence may be selected for download as an MS Excel, CSV, TSV, JSON, or HTML file. If desired, quantitative analysis of this evidence can be conducted by a user outside of HAWC with this downloaded file.

The HAWC form could be an alternative, extension, or supplement to tools already in use by conservation evidence practitioners (Kohl et al., 2018; Supporting Information S6). Web-based literature review management has become a commercial business, with tools that can support the entire process of searching, screening, and evidence extraction (e.g., DistillerSR) or tools that support parts of the process (e.g., SWIFT-Active Screener for screening, Covidence for screening and evidence extraction)—for a fee. For evidence extraction, the HAWC form provides a cost-free, web-based alternative to commercial software and one that is tailored to the unique structure of ecological studies and evidence. The relational database schema underpinning the form is a functional alternative to commercial, user-designed relational databases and coding forms like Knack (https://www.knack.com). The HAWC form could also bridge a gap between open source tools like Colandr (Cheng et al., 2018), which enables screening, and EviA-tlas (Haddaway et al., 2019), which primarily supports visualization of evidence from reviews and evidence mapping activities. Finally, the HAWC form and its visualization capabilities could supplement conservation planning tools like Miradi (https://www.miradishare.org/ux/home) and one-time evidence capture spreadsheets, particularly in situations where synthesizers may want to reuse evidence and decision-makers highly value FAIR principles including documentation and web-accessibility (Salafsky et al., 2022).

The HAWC form has several important limitations. First, the form was built to accommodate evidence extraction from several common ecological study designs, including observational studies, experimental studies in which treatments are applied at multiple levels, and some types of modeling or simulation studies. However, complicated multivariate study designs and analysis approaches, for example, in which causes are characterized as many combinations of interacting factors, pose a challenge for the current version of the form. A more appropriate use of the form will likely be when the cause constitutes a focal intervention type or when the assessment goal is to isolate the effect(s) of a single, discrete pollutant or management action. Second, the form can support a range of qualitative and quantitative assessment and synthesis types, but it currently lacks several features that are desirable for systematic reviews (Collaboration for Environmental Evidence, 2022). For instance, most systematic review guidelines include a process for ensuring consistency across individuals by reconciling independently extracted evidence from a single publication. Currently, HAWC does not allow dual extraction with conflict resolution (e.g., two users extract the same content blindly from a publication as a validation check). Instead, we recommend having a single extractor and then a QA/QC review by an additional person. In addition, the form does not have a part in which to extract details related to internal study validity, although this may be included in future versions.

The US EPA supports the development of HAWC, which is an open-source application (https://github.com/usepa/hawc/) with an MIT (Massachusetts Institute of Technology) License. In addition, US EPA maintains a deployment of the application, US EPA HAWC (https://hawc.epa.gov), used as a compendium for US EPA assessments. The case study linked above (i.e., ORD Assessment Ecological Forms) is one of many assessments that the public can view that demonstrate US EPA HAWC’s capabilities. Note that only US EPA staff can create assessments using US EPA HAWC. However, since the application is open-source, there are other deployments available that allow the public to get an account, develop assessments, and use the form, including https://hawcproject.org (not affiliated with EPA); tutorials on how to use this deployment and additional resources are available on this website.

## 4 | CONCLUSIONS

We envision several applications of the HAWC form by conservation and environmental evidence synthesizers and users. First, teams of evidence synthesizers may find the web-based form useful for collaboration and consistency in evidence extraction, especially if colleagues are spread across multiple organizations. Data exports, visualization exports, or links to HAWC assessments can be easily shared as a part of publications (e.g., figures or supplemental tables) or directly shared with funders and other users to support the accessibility and transparency of projects. The types of questions that conservation evidence synthesizers and users ask include causal questions (e.g., does X cause Y?), efficacy questions (e.g., what is the efficacy of X intervention?), and hypothesis, claim, or assumption-testing questions (e.g., is intervention X is more likely to be effective than intervention Y?). The form, paired with data analysis software, enables synthesizers to answer these types of qualitative and quantitative questions. Public assessments allow evidence users to interact with information and data extracted from studies in ways that are customizable to their needs.

There are several priorities for future updates to the form’s capabilities. First, we recognize that our controlled terminology lists, especially for cause and effect fields, may not serve all potential users of the form. Therefore, we are exploring the possibility of assessment-specific term additions that could be submitted to a term curator. Second, we are interested in adding features to the form that would help users conform to several common systematic review standards, such as multiple evidence extractors for a single publication and a module to extract information related to study quality. Finally, we would like to increase available standard visual outputs, including a standard forest plot to enable efficient understanding, interpretation, and communication of results.

## Supplementary Material

Supplement1

Supplement2

Supplement3

Supplement4

Supplement5

Supplement6

## Data Availability

Source code available and MIT licensed at https://github.com/usepa/hawc.
