# Supplementary material for: Web-based ecological evidence entry form enables consistent, accessible extraction and visualization for synthesis applications
Source: Conserv Sci Pract. Author manuscript; Available in PMC 2026 Jan 23. (PMC11960734; doi:10.1111/csp2.13278)

**Supplemental Information S2.** A simplified database schema for the form. This figure shows the relational nature of the database that underlies the form in a format that will be familiar to database developers. The Field and Type columns in this figure correspond the “Field” and “Format” column in the “Fields and descriptions” tab of Supplemental Information S3. For those that are not database developers and/or are not familiar with this format, please refer to Figure 1 and Supplemental Information S3 for explanation of the form and its parts.


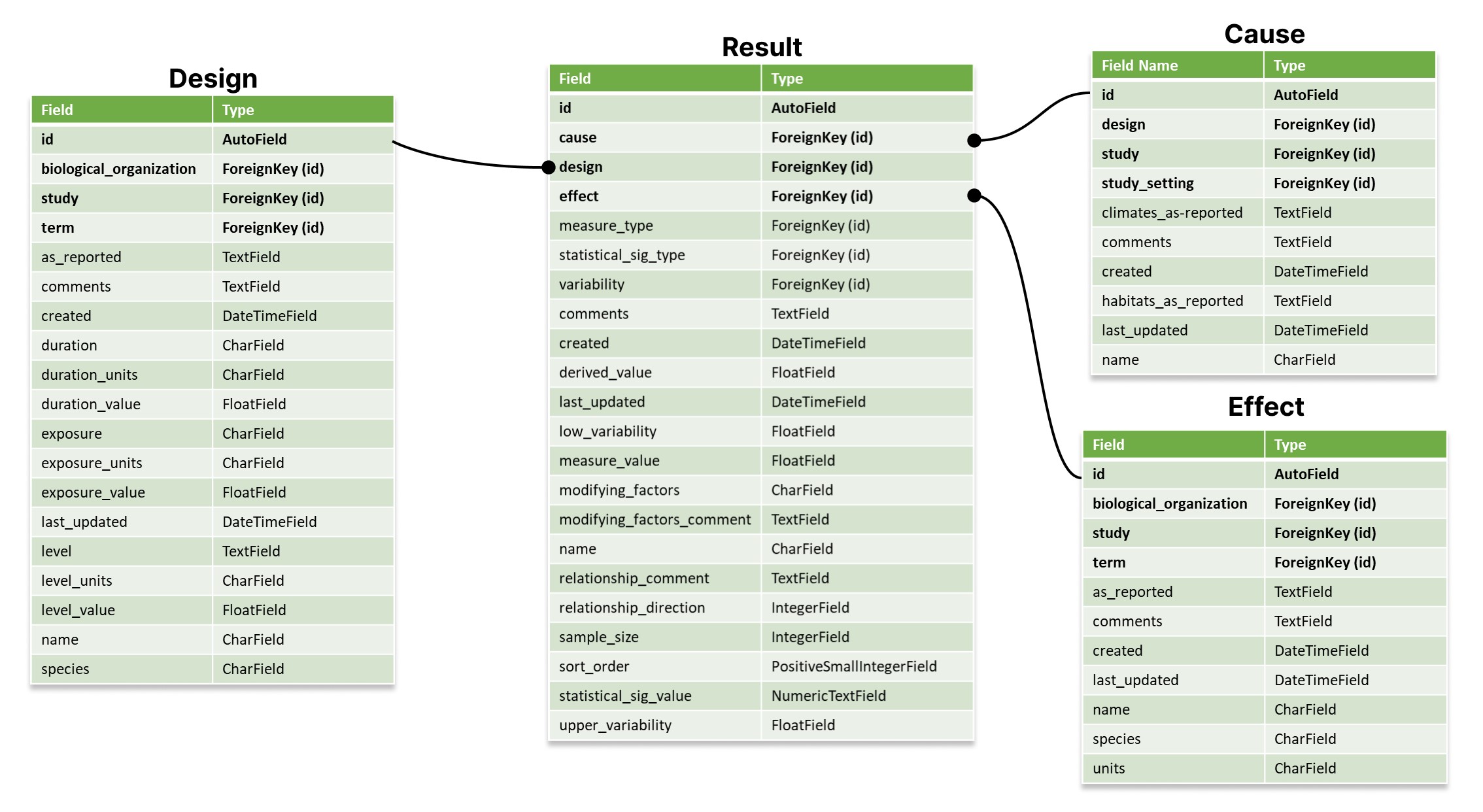

Supplement: Supplement1 [file NIHMS2058004-supplement-Supplement1.docx]
