# Supplementary material for: Web-based ecological evidence entry form enables consistent, accessible extraction and visualization for synthesis applications
Source: Conserv Sci Pract. Author manuscript; Available in PMC 2026 Jan 23. (PMC11960734; doi:10.1111/csp2.13278)

**Supplemental Information S5.** Additional standard heat maps that HAWC creates using evidence extracted into the form. The first heat map summarizes the counts of cause terms across all studies in an assessment. The second heat map summarizes the counts of effect terms across all studies in an assessment.


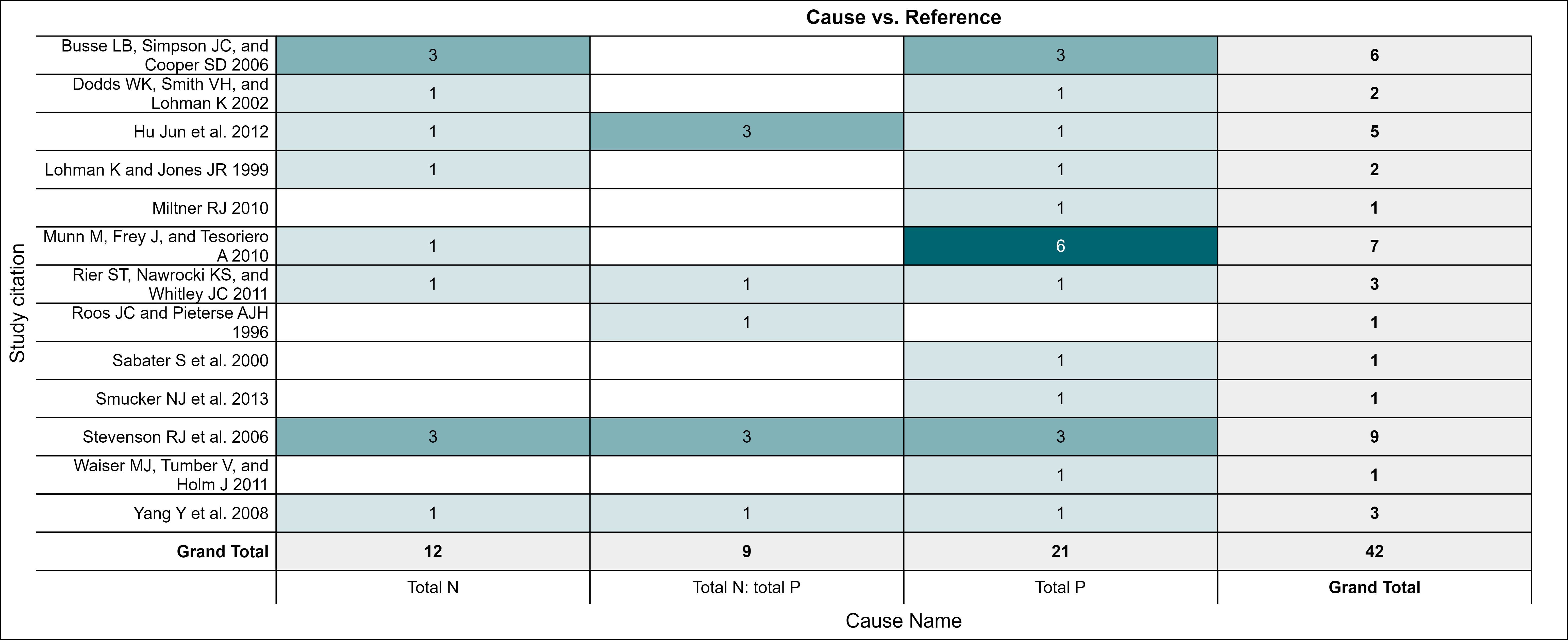

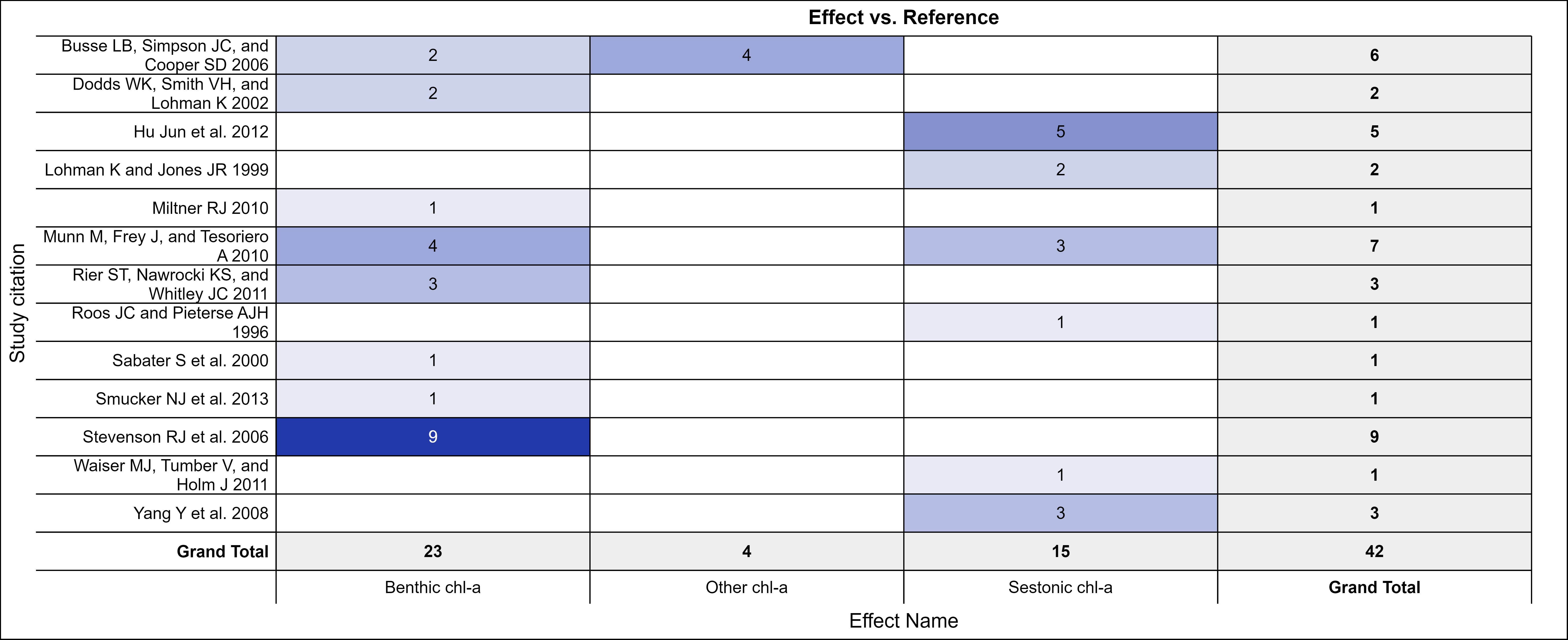

Supplement: Supplement2 [file NIHMS2058004-supplement-Supplement2.docx]
