# Supplementary material for: Web-based ecological evidence entry form enables consistent, accessible extraction and visualization for synthesis applications
Source: Conserv Sci Pract. Author manuscript; Available in PMC 2026 Jan 23. (PMC11960734; doi:10.1111/csp2.13278)
Supplement: Supplement3 [file NIHMS2058004-supplement-Supplement3.docx]

**Supplemental Information S1.** Articles used to test the form.

Gray et al. 2019. Modelling lake cyanobacterial blooms: Disentangling the climate-driven impacts of changing mixed depth and water temperature. Freshwater Biology 64:2141-2155.

Justus et al 2010. A comparison of algal, macroinvertebrate, and fish assemblage indices for assessing low-level nutrient enrichment in wadeable Ozark streams. Ecological Indicators 10.

Mauro et al. 2018. An exploratory study of potential As and Pb contamination by atmospheric deposition in two urban vegetable gardens in Rome, Italy. JOURNAL OF SOILS AND SEDIMENTS. 18 (2). 426-430. 10.1007/s11368-016-1445-y.

Renwick et al. 2008. Water quality trends and changing agricultural practices in a midwest US watershed, 1994-2006. Journal of Environmental Quality 37:1862-1874.

Simkin, S. M., et al. 2016. Conditional vulnerability of plant diversity to atmospheric nitrogen deposition across the United States. Proceedings of the National Academy of Sciences of the United States of America 113(15): 4086-4091.

Sultana et al. 2019. Comparison of water quality thresholds for macroinvertebrates in two Mediterranean catchments quantified by the inferential techniques TITAN and HEA. Ecological Indicators 101.

Szantoi et al. 2009. Cutleaf coneflower (*Rudbeckia laciniata* L.) response to ozone and ethylenediurea (EDU). Environmental Pollution, 157(3), 840-846.

Talhelm et al (2012). Long-term leaf production response to elevated atmospheric carbon dioxide and tropospheric ozone. Ecosystems, 15(1), 71-82.

Zavaleta, E. S., et al. (2003). Additive effects of simulated climate changes, elevated CO2, and nitrogen deposition on grassland diversity. Proceedings of the National Academy of Sciences of the United States of America 100(13): 7650-7654.

Zhang et al 2015. The tolerance of growth and clonal propagation of *Phragmites australis* (common reeds) subjected to lead contamination under elevated CO 2 conditions. RSC Advances, 5(68), 55527-55535.
