# Supplementary material for: Web-based ecological evidence entry form enables consistent, accessible extraction and visualization for synthesis applications
Source: Conserv Sci Pract. Author manuscript; Available in PMC 2026 Jan 23. (PMC11960734; doi:10.1111/csp2.13278)

**Supplemental Information S6.** A generalized workflow of an evidence synthesis process and some of the software that can potentially be used to support the process. This figure is meant to be illustrative. It does not attempt to catalogue all the software tools available to and used by conservation evidence practitioners nor does it attempt to capture all of the features of these tools (e.g., whether or not there is a fee to use). *Evidence Extraction may or may not include an assessment of study quality.


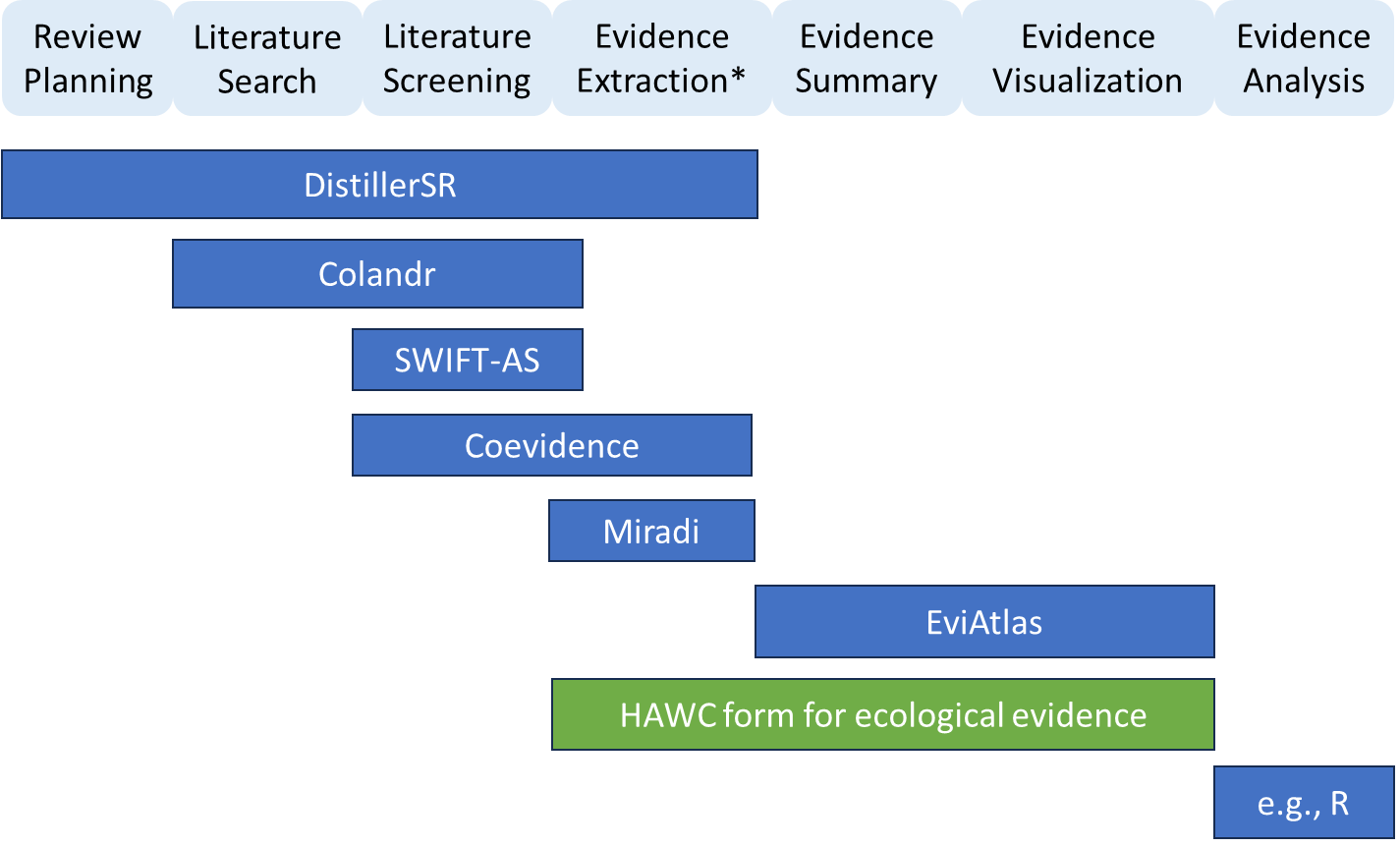

Supplement: Supplement5 [file NIHMS2058004-supplement-Supplement5.docx]
