# Supplementary material for: Web-based ecological evidence entry form enables consistent, accessible extraction and visualization for synthesis applications
Source: Conserv Sci Pract. Author manuscript; Available in PMC 2026 Jan 23. (PMC11960734; doi:10.1111/csp2.13278)

**Supplemental Information S4.** Screenshots of the user interface for the Study Design, Cause, Effect, and Result parts of the form.

Study Design:


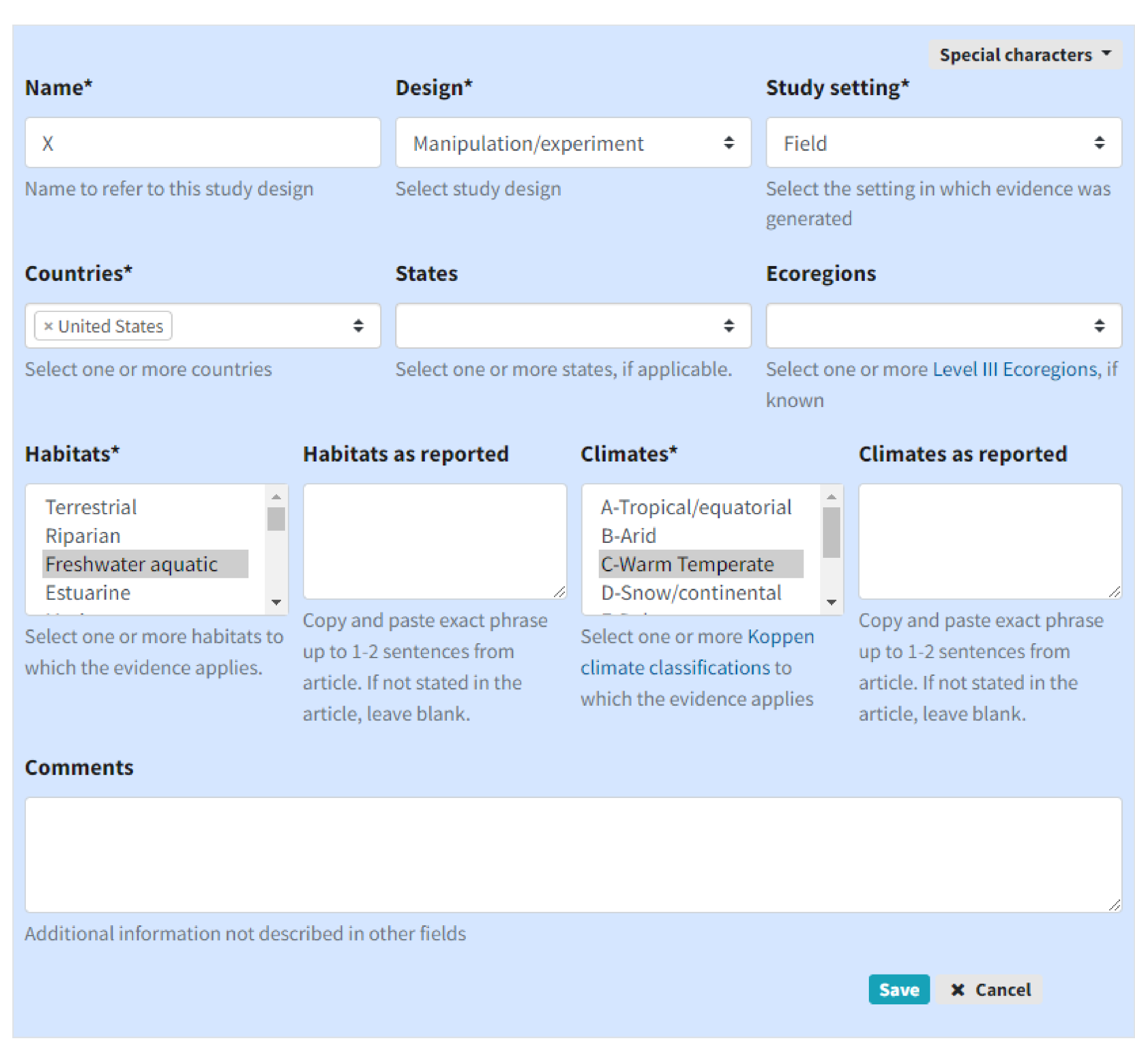


Cause:


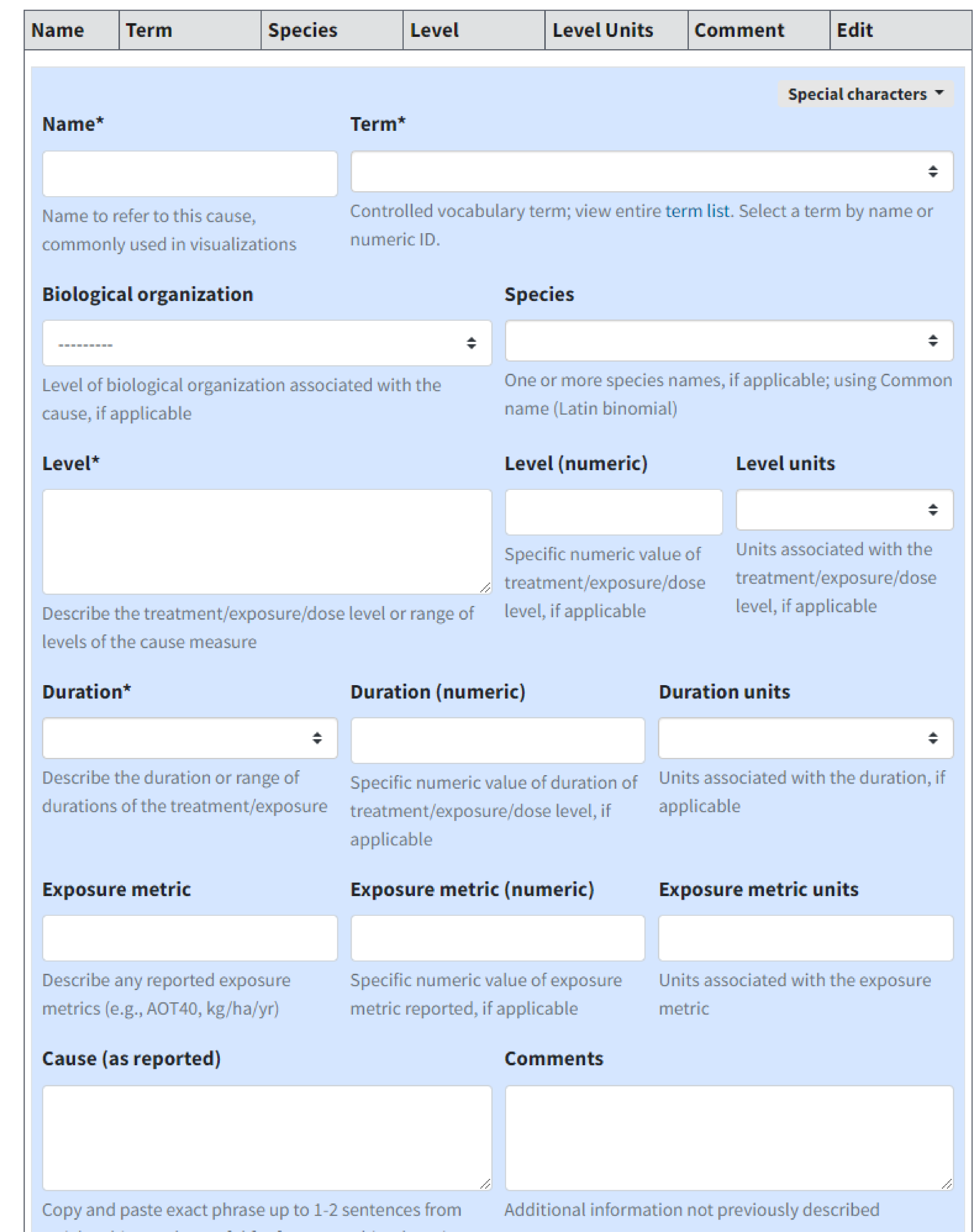


Effect:


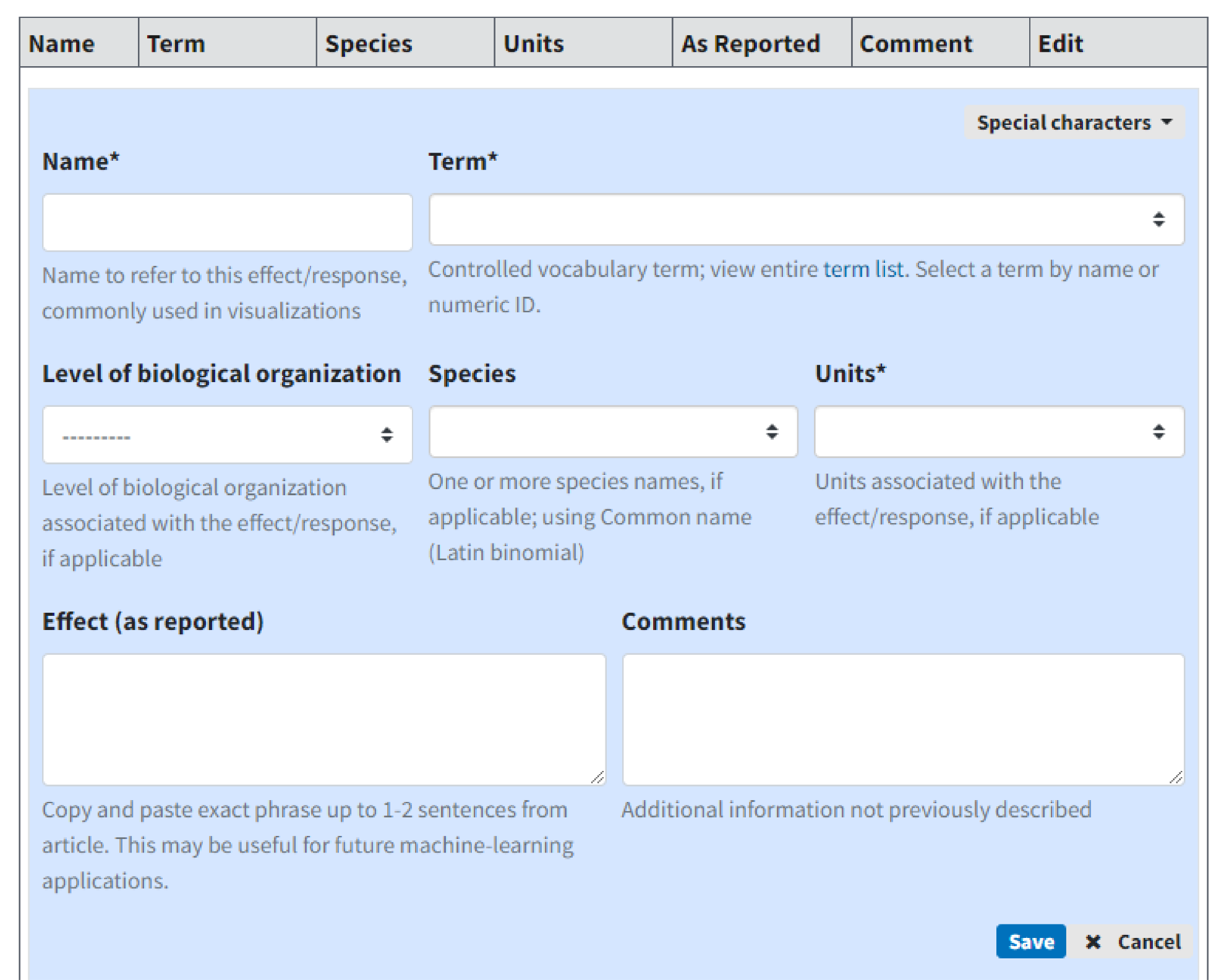


Result:


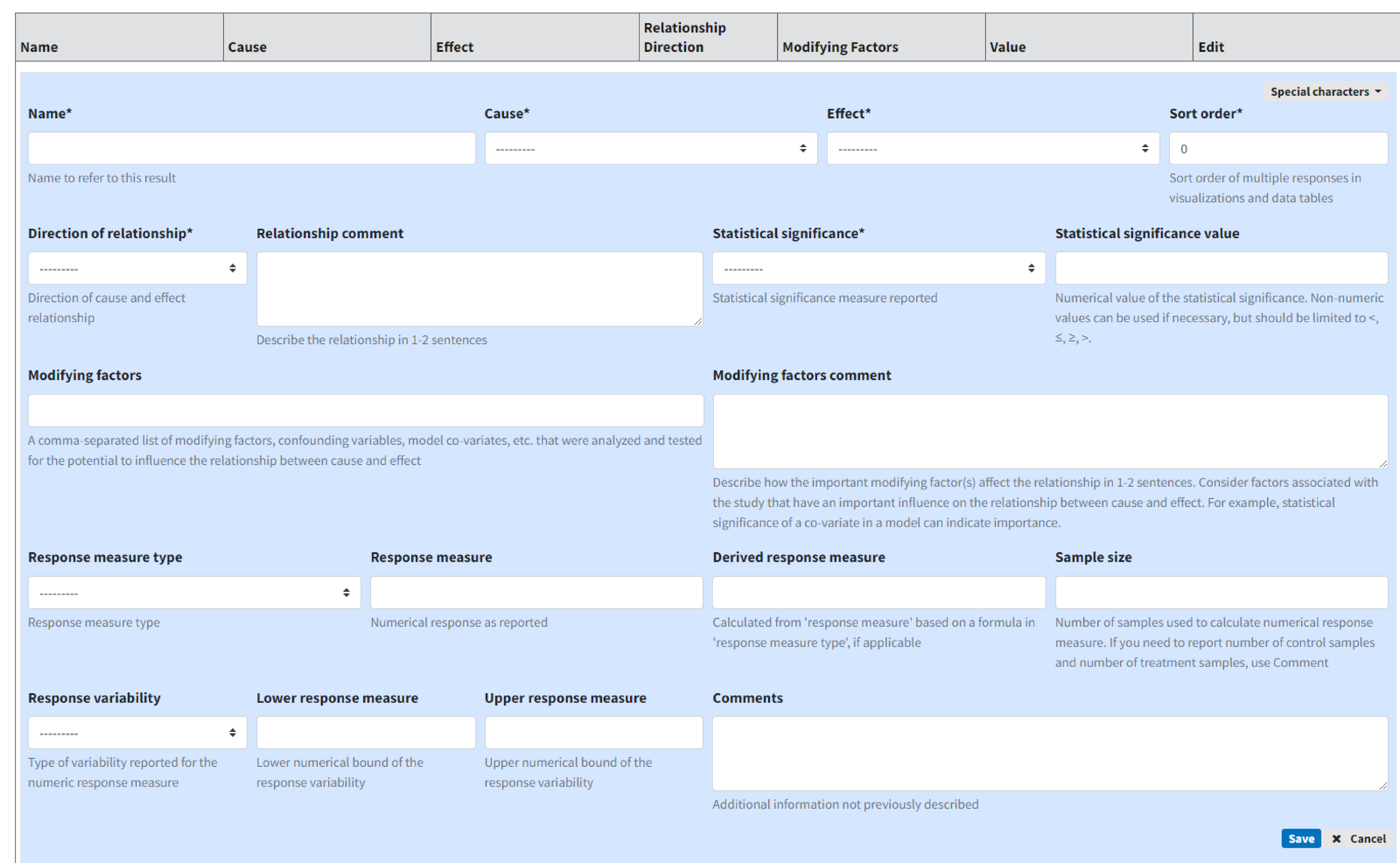

Supplement: Supplement6 [file NIHMS2058004-supplement-Supplement6.docx]
